# A disulfide constrains the ToxR periplasmic domain structure, altering its interactions with ToxS and bile-salts

**DOI:** 10.1038/s41598-020-66050-5

**Published:** 2020-06-02

**Authors:** Charles R. Midgett, Rachel A. Swindell, Maria Pellegrini, F. Jon Kull

**Affiliations:** Department of Chemistry, Dartmouth College, Hanover, NH USA

**Keywords:** Membrane proteins, Transcription factors, NMR spectroscopy, X-ray crystallography, Bacterial toxins

## Abstract

ToxR is a transmembrane transcription factor that, together with its integral membrane periplasmic binding partner ToxS, is conserved across the Vibrionaceae family. In some pathogenic *Vibrios*, including *V. parahaemolyticus* and *V. cholerae*, ToxR is required for bile resistance and virulence, and ToxR is fully activated and protected from degradation by ToxS. ToxS achieves this in part by ensuring formation of an intra-chain disulfide bond in the C-terminal periplasmic domain of ToxR (dbToxRp). In this study, biochemical analysis showed dbToxRp to have a higher affinity for the ToxS periplasmic domain than the non-disulfide bonded conformation. Analysis of our dbToxRp crystal structure showed this is due to disulfide bond stabilization. Furthermore, dbToxRp is structurally homologous to the *V. parahaemolyticus* VtrA periplasmic domain. These results highlight the critical structural role of disulfide bond in ToxR and along with VtrA define a domain fold involved in environmental sensing conserved across the Vibrionaceae family.

## Introduction

ToxR is the founding member of a group of transmembrane transcription factors^1^ and is conserved throughout the gram-negative Vibrionaceae family of marine bacteria^2–5^. Several *Vibrio* species use ToxR to mediate bile resistance^5^, and a few pathogenic species have co-opted ToxR for virulence induction^6,7^. *Vibrio parahaemolyticus* ToxR is important for colonization and virulence in mouse models^7^, where it induces type III secretion system expression by working with VtrA to activate expression of VtrB^7^. ToxS, an integral membrane periplasmic binding partner of ToxR, is also important for *V. parahaemolyticus* colonization in a mouse model^7^. In *V. cholerae*, ToxR is required for human colonization^8^, most likely through induction of bile resistance^5^ and, in conjunction with TcpPH, virulence gene expression^9^.

ToxR regulates many genes in response to stimuli, including *ompT* and *ompU*^10,11^. ToxR directly inhibits *ompT* expression^12^ and activates *ompU* transcription^13^, leading to a change in the outer-membrane porin composition^14^. ToxR is thought to activate virulence by augmenting TcpP activity, leading to *toxT* transcription^9,15,16^ and subsequent expression of cholera toxin and the toxin co-regulated pilus^15,17^. Given the importance of ToxR and ToxS for both bile resistance^5^ and pathogenesis in the *Vibrio* family^7,8^, understanding how they interact with each other and respond to the environment is critical for understanding the disease processes and providing insight into how related proteins function in other pathogenic bacteria^18,19^.

Previously, experiments in minimal media have shown *V. cholerae* ToxR activity can be regulated by the amino acid mixture of N, R, E and, S (NRES)^20^, or bile salts^20,21^. The NRES mixture led to an increase in ToxR and subsequent OmpU expression^20,22^. In contrast, bile salts stimulated ToxR activity without increasing protein amounts^21,22^. This demonstrates ToxR activity can be regulated by two different mechanisms, one dependent on the Var/Csr system to increase ToxR mRNA and protein expression^22^, and a second to mobilize existing ToxR to become transcriptionally competent^21,22^. We hypothesize this potentially occurs via bile salts interacting with the ToxR periplasmic domain.

While over-expression of ToxR can lead to ToxR regulon transcription, under physiological conditions ToxR requires ToxS, an integral membrane protein with a periplasmic domain, for full activity^23^. In addition, ToxS protects ToxR from degradation in conditions of stationary growth and alkaline pH^24,25^. Furthermore, ToxS is known to affect the formation of a disulfide bond in the ToxR periplasmic domain^26,27^. Investigations into the contribution of cysteines C236 and C293 on *V. cholerae* ToxR function showed single cysteine mutants were inactive^28^. However, as this paper was being prepared a report was published showing when ToxR C293S was overexpressed it formed a disulfide linked homo-dimer active in porin regulation in a Δ*degP* background^29^. Whereas a double mutant had decreased function in porin regulation, but no defects in virulence gene expression^27^. Moreover, the double mutant was shown to be more susceptible to degradation than wild-type ToxR, even in the stabilizing presence of ToxS^25^. In wild type ToxR, these cysteines are known to form either an intra-chain disulfide bond or a disulfide linked homodimer, which is present when *toxS* is deleted in *V. cholerae*^26,27^. Since *ΔtoxS* strains have defects in ToxR activity^20,21,27^, it follows that the intra-chain disulfide bonded, monomeric form of the ToxR periplasmic domain is the physiological active conformation.

While the intra-chain disulfide bonded conformation of ToxR is certainly the predominant form^26,27^, ToxR lacking a disulfide bond has been hypothesized to be present *in vivo* based on two prior studies. First, the periplasmic domain of ToxR and TcpP can form a disulfide linked heterodimer^28^, which can only occur if the two cysteines in ToxR are free to form a bond. Second, bile salts induce disulfide stress by inhibiting the activity of DsbA^30^, the chaperone responsible for inducing disulfide bond formation^31^. Prior work in our laboratory showed the non-disulfide bonded ToxR periplasmic domain was destabilized by bile salts, however, the salts increased binding to the ToxS periplasmic domain (ToxSp). This led us to hypothesize the ToxR periplasmic domain has evolved to become destabilized by bile salts, leading to stronger ToxS binding and increased activity of ToxR^21^.

To characterize and visualize the differences between the disulfide bonded and non-disulfide bonded forms of ToxR we expressed and purified the disulfide bonded conformer of the ToxR periplasmic domains (dbToxRp) from *V. cholerae* and *V. vulnificus* for biochemical and structural studies. The dbToxRp was able to bind ToxSp, and was destabilized by bile salts, although, unlike the non-disulfide bonded ToxR periplasmic domain (ToxRp), bile salts did not increase the binding of dbToxRp to ToxSp. The structure of dbToxRp showed the domain is globular composed of 2 α-helices, linked via the disulfide bond, and a β-sheet composed of 5 β-strands. Furthermore, we found that dbToxRp is structurally homologous to the periplasmic domain of VtrA^18^. VtrA is a transmembrane transcription factor that shares similarities with ToxR as both have an OmpR DNA binding domain and a periplasmic domain that is involved in bile sensing^18,32,33^. Overall, these results demonstrate the important role of ToxR disulfide bond formation in the interaction with ToxS. This represents a first step in understanding the structure function relationship for virulence induction and bile resistance, paving the way for a better understanding of the biochemical nature of ToxR activation as well as related proteins^18,19^.

## Results

### ToxR containing a disulfide bond is folded and active

**Figure 1:**
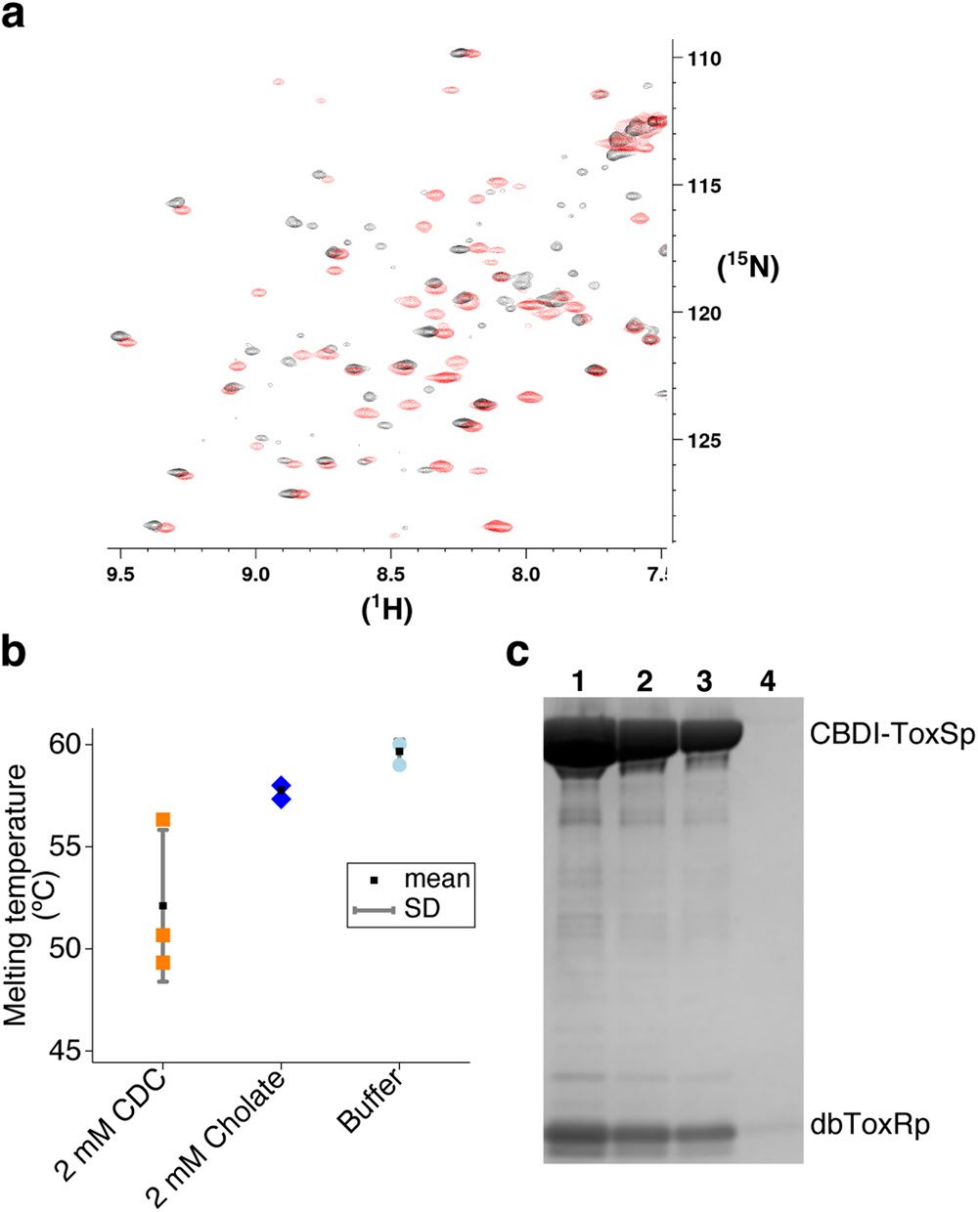
Validation of the dbToxRp construct showing it is similar in structure to ToxRp, destabilized by bile salts, and can bind ToxSp. (**a**) A HSQC of ^15^N-dbToxRp in black overlaid with a HSQC of ^15^N-ToxRp in red. The NMR data was collected on a Bruker 700 MHz instrument. (**b**) Melting temperatures of dbToxRp when treated with 2 mM cholate and chenodeoxycholate as measured by DSF. Each trial is graphed as well as the mean and SD, n = 3. (**c**) A colloidal blue stained gel showing the dbToxRp can be pulled down by CBD-ToxSp. Lane 1–3: serial dilutions 1×-1/4x of the CBD-ToxSp pull down with dbToxRP, Lane 4: dbToxRp with beads only.

The *V. cholerae* disulfide ToxR periplasmic domain (Vc-dbToxRp) construct was confirmed to be structurally and functionally similar to the previously analyzed ToxRp^21^ by comparing NMR^1^H,^15^N HSQC’s, determining if chenodeoxycholate (CDC) and cholate could lower the melting temperature, and assessing its ability to bind to the *V. cholerae* ToxS periplasmic domain tagged with an N-terminal chitin binding domain (CBD-ToxSp) (Fig. 1). A comparison of the Vc-dbToxRp HSQC with the previously collected HSQC of the ToxRp shows the two conformations share a common core fold (Fig. 1a), as demonstrated by a series of well dispersed peaks that overlap in the two conformations. About 10 extra intense peaks are observed for Vc-ToxRp in the central region of the spectrum, consistent with random coil shifts corresponding to the unfolding of the short helix α2. Differential scanning fluorometry in the presence of cholate and CDC showed Vc-dbToxRp was destabilized by the bile salts, with CDC having the greatest effect (Fig. 1b), demonstrating the disulfide bonded conformer was destabilized in a manner similar to the non-disulfide bonded form as previously described^21^. Finally, a pull down showed the Vc-dbToxRp interacted with the CBD-ToxSp (Fig. 1c). Taken together, these tests confirm that Vc-dbToxRp is structurally and functionally similar to Vc-ToxRp.

### dbToxRp shows increased binding to ToxSp, unaffected by bile salts

**Figure 2:**
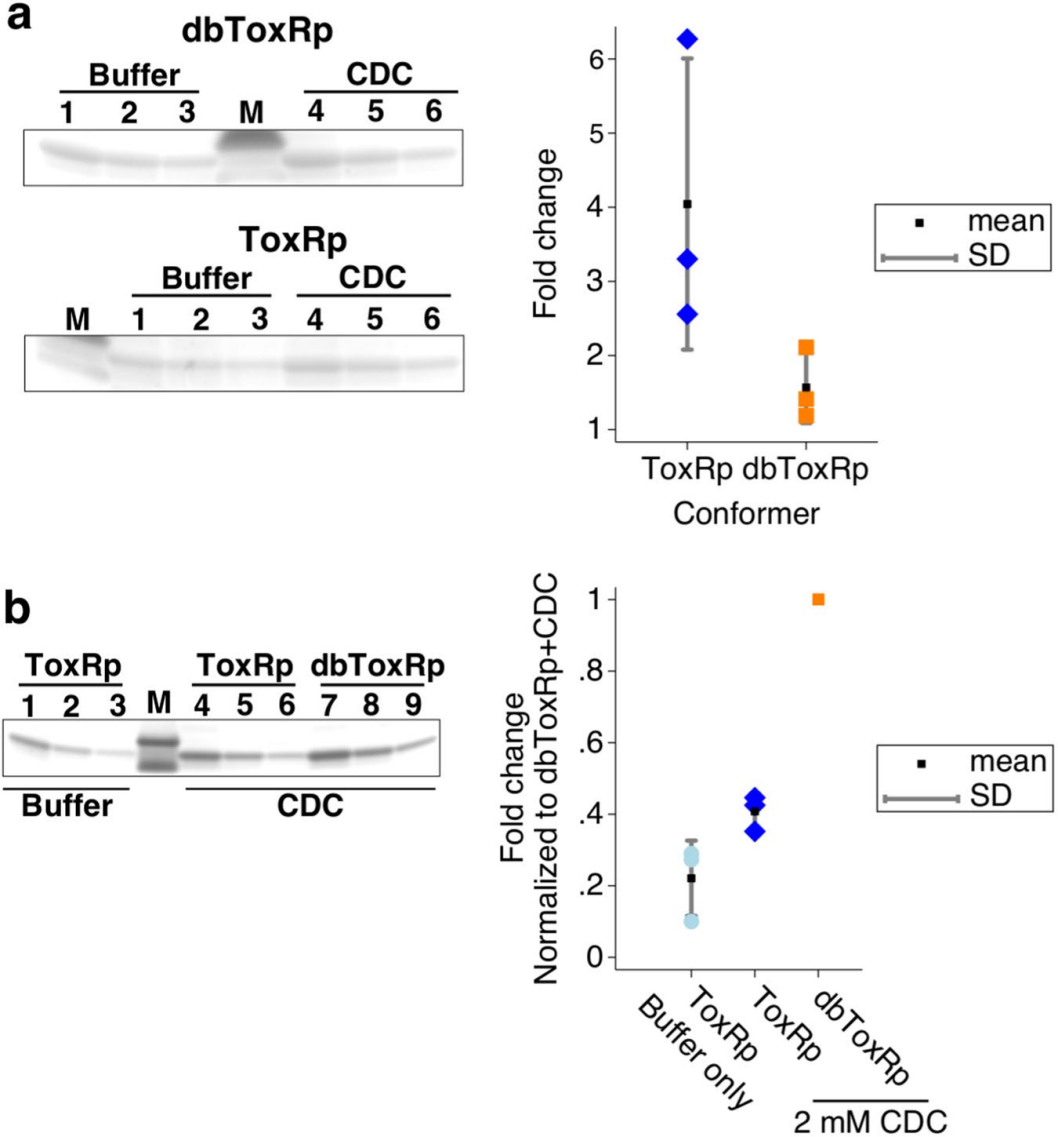
dbToxRp binds to ToxSp 2–4 fold more than ToxRp and the binding is not effected by bile salts. (**a**) Left top panel a colloidal blue stained gel excerpt showing the changes in the amount of dbToxRp pulled down when the domain was treated with buffer versus 2 mM CDC. Left bottom panel a colloidal blue stained gel excerpt showing the changes in ToxRp pulled down when the domain was treated with buffer or 2 mM CDC. Lanes 1–3 serial dilutions of the CDC treated pull down and 4–6 serial dilutions of the pull down with buffer. M is the lane with the marker. Right panel is a graph showing the relative amount of the domains pulled down by the compounds versus buffer. Each trial is represented as well as the mean and SD, n = 3. (**b**) Left panel gel of the periplasmic domains pulled down from the above experiments to determine the relative amount of ToxRp pulled down normalized to dbToxRp. Graph of the results normalized to the pull down performed with dbToxRp + 2 mM CDC (The intensity of dbToxRp with 2 mM CDC 1x dilution band was set to one in each trial). Each trial is represented as well as the mean and SD, n = 3.

Previous work in our laboratory revealed, somewhat unexpectedly, that the bile salt CDC increased the interaction between the periplasmic domains of ToxR and ToxS^21^. To test if CDC also increases the affinity of Vc-dbToxRp to CBD-ToxSp we performed pull downs with ToxRp and Vc-dbToxRp in the presence and absence of CDC. Similar to previous results, CDC increased the amount of Vc-ToxRp pulled down by CBD-ToxSp (fold change of 4.0 ± 2.0). However, CDC had a negligible effect on the amount of Vc-dbToxRp pulled down (fold change of 1.6 ± 0.5), indicating Vc-dbToxRp binding to ToxS is unaffected by bile salts (Fig. 2a). To compare the amounts of the ToxR periplasmic domains pulled down in the above experiments we ran selected pull downs on the same gel to calculate relative amounts. Interestingly, the presence of a disulfide bond increased the interaction between Vc-dbToxRp and CBD-ToxSp. The results clearly showed Vc-dbToxRp with CDC was pulled down more than Vc-ToxRp in the presence or absence of CDC. Quantification of the relative amounts pulled down showed Vc-ToxRp was pulled down to a relative level of 0.22 ± 0.11 to that of Vc-dbToxRp with CDC, and Vc-ToxRp with CDC was pulled down to a relative level of 0.41 ± 0.05. (Fig. 2b). These results suggest the presence of a disulfide bond in the ToxR periplasmic domain significantly increases the affinity of ToxR for ToxS.

### Crystal structures reveal the structural role of the ToxR periplasmic domain disulfide bond

**Figure 3:**
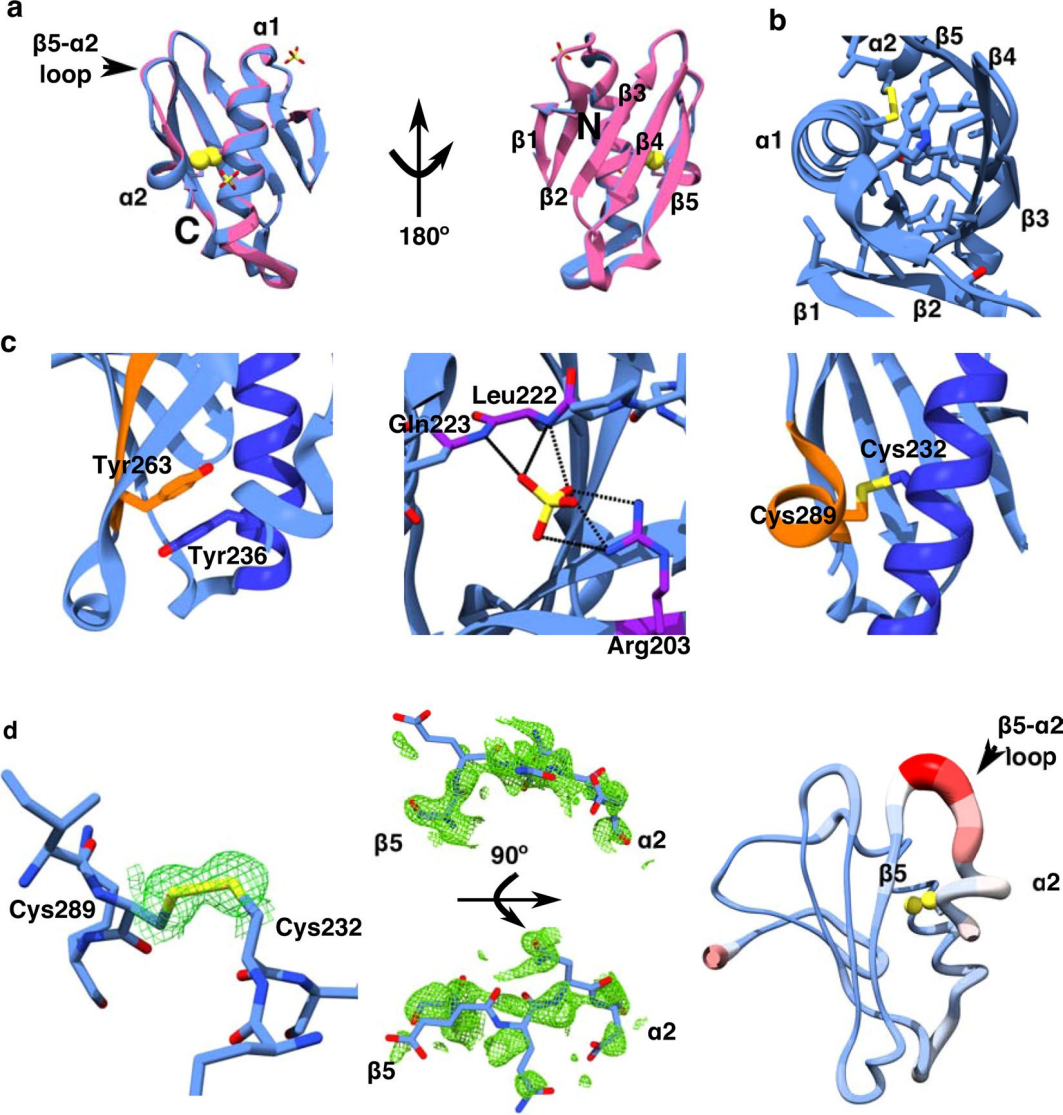
The structure of the *V. vulnificus* dbToxRp showing selected interactions, the α2-β5 loop disorder, and structural homology with the VtrA periplasmic domain. (**a)** Alignment of the SeMet-Vv-dbToxRp (pink) with the native Vv-dbToxRp (blue performed with Chimera using MatchMaker). Sulfate ions in the asymmetric units of the SeMet and native structures are shown as sticks colored by atom type, and the cysteine sulfur atoms are depicted as yellow spheres. From left to right; the helix face of the structure, followed by the sheet face of the structure. The secondary structure elements, the β5-α2 loop, as well as the N and C termini are labeled. (**b**) A “top” view showing the hydrophobic interactions packing the helices to the β-sheet with the secondary structure elements labelled. **c**. Close up of selected interactions in the Vv-dbToxRp structure. Left to right; Pi-Pi stacking of Tyr236 on α1 (blue) and Tyr263 on β4 (orange) in the hydrophobic core. The sulfate ion interactions with side chain of Arg203 along with the amides of Leu222 and Gln223 (purple). The dotted lines show the H-bonds between the protein and sulfate ion. The disulfide bond (yellow) that attaches α2 (orange) to α1 (blue) through Cys289 and Cys232. (**d)** Left to right; the composite-omit map (2Fo-Fc) density surrounding the disulfide bond displayed as green mesh contoured to 1σ. The composite-omit map (2Fo-Fc) density around the β5-α2 loop (Ser280-Asp284) is displayed as a green mesh contoured to 1σ with the connections to β5 and α2 labeled. Top panel is a side view and the bottom panel is the top view of the loop. Last panel, representation of the B-factors showing the β5-α2 loop has the highest B-factors in the structure. The cysteine sulfur atoms are displayed as yellow spheres, β5, α2 and the β5-α2 loop are labeled.

Efforts to solve the structure of the *V. cholerae* dbToxRp were unsuccessful necessitating the need to screen ToxR periplasmic domains from several *Vibrio sp*. to find a suitable target for crystallization. The dbToxRp from *V. vulnificus*, which shares 55.2% identity to the *V. cholerae* ToxR periplasmic domain, readily crystallized. The crystal structure of *V. vulnificus* intra-chain disulfide ToxR periplasmic domain (Vv-dbToxRp) was solved by molecular replacement using a selenomethionine (SeMet) labeled structure to 1.25 Å (Statistics are shown in Table S1). The SeMet and native structures were aligned in Chimera^34^ using MatchMaker^35^ with an RMSD of 0.324 Å over the backbone of all 91 resolved residues (Fig. 3a). Given the two structures are essentially the same we focused on analyzing the native structure. The disulfide bonded ToxR periplasmic domain consists of 5 β-strands and 2 α-helices forming an α/β fold. Topologically the domain starts with the first two anti-parallel β-strands followed by the first α-helix. This is followed by the last 3 β-strands, and the structure terminates with the second α-helix which forms a disulfide with α1. The 5 β-strands are arranged in a β-sheet with one side packed against the two α-helices, through hydrophobic interactions (Fig. 3b). The other side of the sheet is solvent exposed. Interactions of note include a Pi-Pi stacking interaction between the Tyr236 from α1 and Tyr263 from β4 (Fig. 3c left panel) in the hydrophobic core of the protein. A sulfate ion was found in the structure, stabilized by the side chain of Arg203 and the amide nitrogens of Leu222 and Gln223 (Fig. 3c middle panel). Finally, the disulfide bond between Cys232 and Cys289 covalently attaches α2 to α1 stabilizing the structure (Fig. 3c left panel). The structure is well ordered, as typified by the clearly visible electron density for the disulfide bond in a composite omit map of the structure (Fig. 3d first panel). Interestingly, the density of the α2-β5 loop (residues 281–284) was poorly defined (Fig. 3d middle panel). In addition, the α2-β5 loop has the highest B-factors in the structure (Fig. 3d last panel). Furthermore, standardization (Z-score) of the Cα B-factors showed the 281–284 Cα Z-scores are in the top 5% of the distribution (Fig S1). Taken together this indicates the loop is flexible. Therefore, we hypothesize a major role of the disulfide bond is to stabilize the structure, and importantly the ToxS binding region, by constraining this flexible loop as well as the relatively short helix, α2, which follows it.

### VtrA and ToxR periplasmic domains are structural and functional homologues

The structure of the Vv-dbToxRp was similar to the previously published structure of the VtrA periplasmic domain from *V. parahaemolyticus*^18^. VtrA, like ToxR, is a transmembrane transcription factor with a winged helix-turn-helix domain and a C-terminal periplasmic domain involved in virulence induction in response to bile salts^32,33^, potentially by sensing them with its binding partner VtrC^18^. However, to take an unbiased approach to find ToxR periplasmic domain structural homologs we performed a DALI search^36^. The search returned a number of proteins with various functions generally unrelated to the function of ToxR. Interestingly, VtrA was listed as the 118^th^ result that matched the dbToxRp. To gain more insight we curated the list to remove potential duplicated proteins, assessed the cellular location of the proteins (extracellular, cytoplasmic, periplasmic, etc.), and determined if any contained disulfide bonds for the top 50 proteins, in which VtrA ranks 38. Only 4 other periplasmic proteins were identified, and 8 extracellular proteins. Out of those only 5 had disulfide bonds, and none of the disulfide bonds are in comparable areas as the dbToxRp bond (Fig S2). Given the above results and the low percent identity of the hits returned from the DALI server we further examined the structures using Chimera^34^. The pdb files of the structures up to the VtrA periplasmic domain were downloaded from the PDB and aligned to the Vv-dbToxRp structure using MatchMaker^35^, with the secondary structure weighted at 95%. Interestingly, MatchMaker^35^ aligned the VtrA periplasmic domain to dbToxRp with the lowest all atom RMSD of 5.5 Å (Table S2), suggesting despite the placement of VtrA in the DALI search the VtrA and ToxR periplasmic domains are structural homologs.

**Figure 4:**
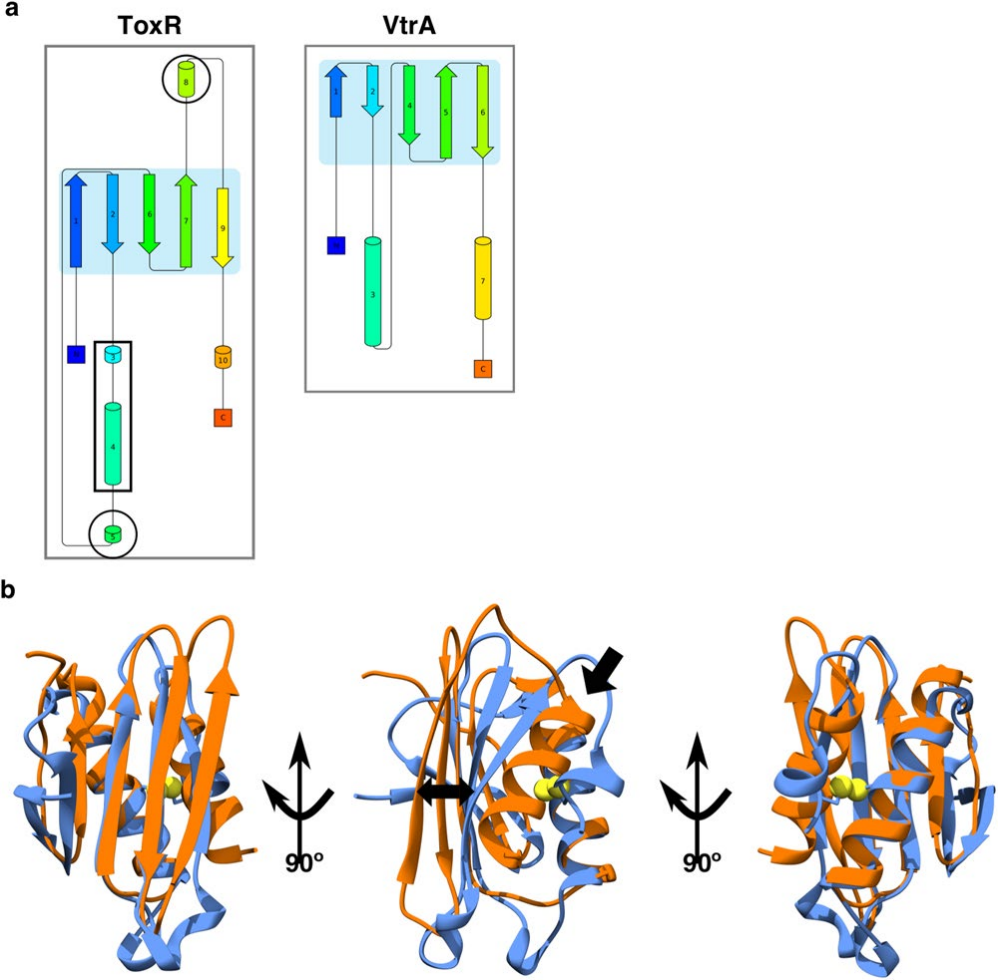
VtrA and ToxR periplasmic domains are structural homologs. (**a**) Shows topological graphs of the ToxR and VtrA periplasmic domains generated by Pro-origami. The graph secondary structure elements are colored from N-terminal (blue) to C-terminal (red). The shaded area is the core domain as determined by Pro-origami. While Pro-origami assigned some of the loop regions in ToxR as helices (circled) and α1 in ToxR was broken into two helices (rectangle) the two structures share the same topology. **(b**) Overlay of the VtrA structure, (5kev) (orange) and the Vv-dbToxRp structure (blue). A single arrow points to the VtrA terminal helix, (middle panel) and a double arrow shows the displacement of the last strand between the VtrA and ToxR (middle panel). The cysteine sulfur atoms are shown as yellow spheres for clarity.

Given the structural homology between the domains, we examined the alignment of the two structures in Chimera, using the default parameters^34^, as well as assessed topological plots produced by Pro-origami^37^. The alignment and plots showed the domains have the same topology, with all secondary structure elements in similar positions. Both have the N-terminal two β-strands followed by the first helix, then the next three β-strands completing the β-sheet, and finally terminating with the second helix (Fig. 4). The main difference between the two structures is the position of the last β-strand and α-helix. In VtrA the last helix is longer pushing the last β-strand further away from the core structure (Fig. 4b middle panel). Also, as the periplasmic domain of VtrA does not contain cysteines (Fig. 4b last panel) there can be no stabilizing disulfide bond. However, based on the clear topological, structural, and functional similarities we propose the periplasmic domains of ToxR and VtrA represent a domain family utilized in sensing the extra-cellular environment.

### dbToxRp lacks a dimerization interface

The periplasmic domain of ToxR has been proposed to mediate dimerization, which is thought to be essential for activity. Prior studies replacing the periplasmic domain of ToxR with proteins known to dimerize, resulted in active constructs^38–41^. Therefore, we assessed the ability of the purified disulfide bonded ToxR periplasmic domain to form dimers *in-vitro* and in the crystal. To assess the ability of the domain to dimerize in solution we performed analytical ultracentrifugation. The domain sedimented as a single species with a molecular weight of 11.8 kDa and 12.7 kDa, consistent with a monomer (Fig S3) of predicted molecular weight of 11.5 kDa. In the crystal the *V. vulnificus* dbToxRp made two contacts whose biological relevance were assessed using PISA^42^. Both contacts were determined to be due to crystal packing (Fig S4). Therefore, we conclude dbToxRp is most likely monomeric.

## Discussion

Here we have biochemically characterized and solved the structure of the ToxR periplasmic domain with an intra-chain disulfide bond. This conformation was found to be similar to the non-disulfide conformation as confirmed by NMR, indicating both constructs consist of a stable core fold. In addition, the disulfide conformer is destabilized by bile salts, similar to previously published results^21^. However, in contrast to ToxRp^21^, bile salts had no effect on the binding of the dbToxRp conformer to ToxSp. Furthermore, dbToxRp bound ToxSp about 2–4 fold more in our pull down assay than the non-disulfide bond conformer, suggesting the disulfide bond stabilizes the ToxS binding interface.

**Figure 5:**
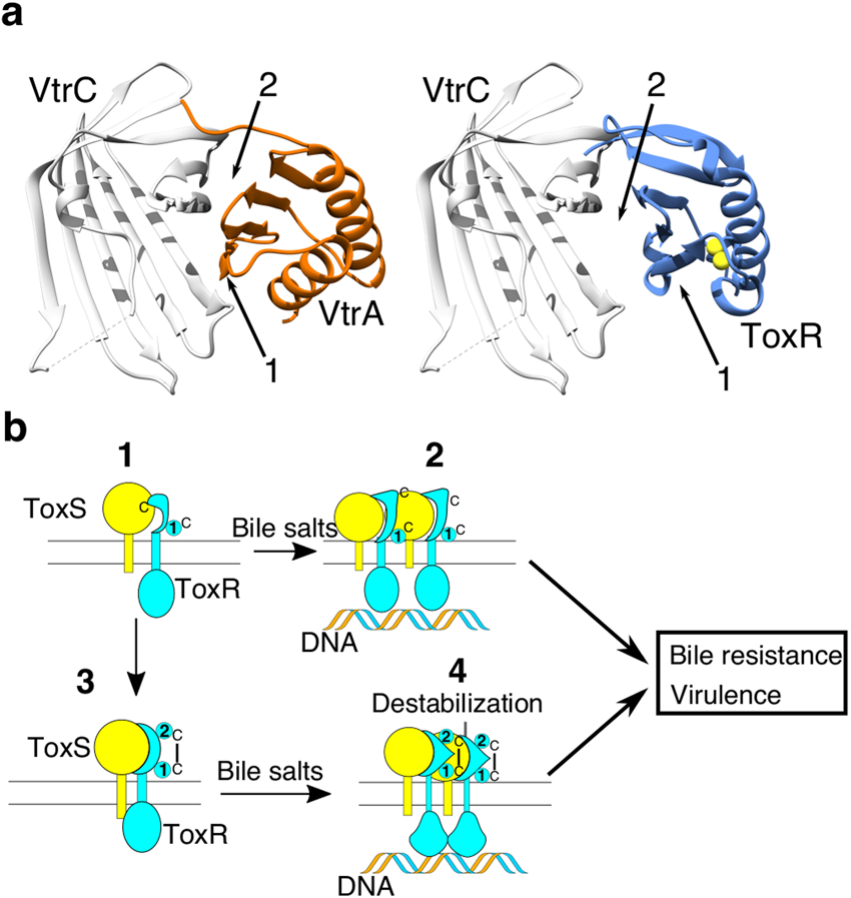
Models of potential interactions between ToxR and ToxS periplasmic domain using the VtrAC structure, and how ToxR and ToxS interact to induce virulence as well as bile resistance. (**a**) The left panel shows the VtrAC (5kev) (VtrA orange, VtrC in gray) structure with arrows pointing to the interfaces between the proteins. Arrow 1 points to the VtrA β5-strand that extends the VtrC β-sheet, and arrow 2 points to VtrA β-sandwich interaction with VtrC. In the right panel the Vv-dbToxRp structure (blue) was aligned with VtrA in the VtrAC structure. VtrC (gray) is presented as a stand in for ToxS. The numbered arrows point to the β5 strand (1) and the potential β-sheet interface (2). (**b**) A model of the potential interactions between ToxR, ToxS, and bile salts. ToxR is in blue with the helices depicted as circles and numbered, ToxS is in yellow, and DNA is represented by an orange and blue helix. The non-disulfide bonded form of ToxR, with only helix1, interacts weakly with ToxS (1). However, bile salts increase the interaction of the non-disulfide bonded ToxR to ToxS, leading to ToxR binding DNA allowing for ToxR periplasmic domain dimerization and activation (2). In addition, ToxS favors the formation of the ToxR intra-chain periplasmic disulfide bond, which increases the affinity between ToxR and ToxS (3). In this case bile salts destabilize the periplasmic domain, which remains bound to ToxS. The destabilization is transmitted across the membrane to the DNA binding domain allowing for transcriptional activity, augmented by ToxS (4). Again, ToxR binding to ToxS and DNA allows for ToxR periplasmic domain interactions.

The dbToxRp structure is clearly homologous to the structure of the VtrA periplasmic domain^18^. Furthermore, given VtrA and ToxR share functional similarities we can hypothesize how ToxR and ToxS interact based on what is known about VtrA. Previously, the VtrA periplasmic domain was crystallized with its binding partner the VtrC periplasmic domain. The structure showed the proteins interact in two ways, via parallel beta strand hydrogen bonds between the β5 strand of the VtrA sheet and the most N-terminal β-strand of the VtrC beta-barrel (arrow 1 in Fig. 5a left panel), as well as β-sandwich like interaction between the VtrA β-sheet and the C-terminal region of the VtrC β-barrel (arrow two in Fig. 5a left panel)^18^. Based on this, we predict ToxS is a structural homolog of VtrC and the ToxR periplasmic domain will bind ToxS in a similar manner, as shown by a structural alignment of dbToxRp with VtrAC (Fig. 5a right panel). Despite these structural similarities there are differences between these two regulatory pairs. First, VtrA requires VtrC for expression^18^ whereas ToxR can be expressed by itself^20,21,23,26^. Second, ToxR depends on the formation of an intra-chain disulfide bond to properly fold while the VtrA periplasmic domain is cysteine free. It would be interesting to determine if the ToxR periplasmic domain could be stabilized without a disulfide bond and what effect that would have on ToxR function.

By combining our results with insight gleaned from the VtrAC structure, we can begin to explain how ToxR and ToxS might interact. At least *in vitro*, ToxR can exist in three forms, a monomer with an intra-chain disulfide bond, a disulfide-free monomer, and a (likely non-physiological) disulfide bonded homodimer. Disulfide formation in the ToxR monomer apparently constrains the β5-α2 loop and the adjacent α2 helix. This, based on structural homology with VtrAC, stabilizes the binding interface between ToxR and ToxS. Such an increase in stability is consistent with observations regarding the degradation of the double cysteine mutant in Lembke *et al*. 2018^25^. Interestingly it has been observed that ToxS enhances ToxR intra-chain disulfide bond formation^26^. How this occurs in the context of the above results is unexplored.

The structure of the dbToxRp provides a potential explanation to why breaking the disulfide bond interferes with ToxS binding and how bile salts can modulate the interaction. In the absence of intra-chain disulfide bond formation, ToxR is destabilized and it is unlikely the short α2 helix would form. Additionally, α2 might assume an alternate conformation in which it blocks interaction with ToxS. Given bile salts increase the ToxRp-ToxSp interaction suggests bile salts disrupt whatever alternate structure α2 forms in the absence of the disulfide bond, thereby allowing ToxS binding. In dbToxRp, the loop and α2 are already in a conformation that stabilizes the interface, rather than blocking it, and bile salts have no effect on ToxS binding.

This leaves open the question of how ToxS augments ToxR activity. ToxR is thought to be active as a dimer and its periplasmic domain has been presumed to mediate dimerization^38–41^. While it has been proposed that ToxR cytoplasmic domains may interact in the presence of DNA^29,43^, when the periplasmic domain of ToxR was replaced by MalE, which does not dimerize, ToxR activity decreased, indicating the periplasmic domain mediates dimerization^39,40^. Our biochemical analysis indicates the domain is a monomer in solution and structural analysis failed to find a dimer interface, arguing for other determinants of dimerization. Given our data, and the stipulation the work in this report has been done with purified domains, we propose ToxS is the most likely candidate for this, and the disulfide bond dependent interaction of ToxR with ToxS results in formation of a dimer of ToxR and ToxS dimers, forming a complex competent for ToxR activation. Future structural studies aimed at determining the structures of ToxSp as well as that of the complex between dbToxRp and ToxSp should clarify if this is the mechanism for dimerization.

Alternately, given ToxR binds DNA, DNA could very well provide the impetus for ToxR oligomerization. In such a model ToxR binds DNA, and weak protein-protein interactions could promote dimerization or higher order oligomers, as seen in experiments with VtrA^44^. Yet another possibility is DNA organizes ToxR in the membrane without specific interactions between domains. Determining which might be the case will require reconstruction of full-length ToxR in a membrane and in the presence of DNA.

While the work described here has been performed with isolated domains and require experiments with full-length proteins for confirmation, our results suggest a model of bile salt mediated ToxR activation, in which ToxR is a monomer in the membrane, binding to ToxS. The non-disulfide bonded conformation has a low affinity for ToxS. The interaction between the two proteins increases in the presence of bile salts, potentially by the destabilization of steric clashes. This would lead to increased ToxR binding to DNA, allowing for ToxR-ToxR interactions, ensuring ToxR activity in the face of bile salt induced disulfide stress^45^. Alternately, ToxR without a disulfide bond is the conformation that responds to bile salts. However, a double cysteine to serine mutant showed reduced porin regulation in the face of osmotic stress, although the mutant was still able to regulate virulence production^27^. When ToxR contains an intra-chain disulfide bond, its affinity for ToxS is increased and unaffected by bile salts. Given this form of ToxR is the most abundant, we believe it is involved in bile sensing. When the domain is destabilized by bile salts, this signal is transmitted across the membrane, increasing the ability of ToxR to bind DNA. This effect is augmented by interaction with ToxS, which remains bound to ToxR. In this model, the action of ToxS with DNA allows for the dimerization of ToxR (Fig. 5b). Recently, Lembke et. al. 2020 has suggested the ToxR periplasmic domains may have weak protein-protein interactions that are stabilized by ToxS. Then in the presence of bile salts and DNA the ToxR-ToxS interaction peaks and this allows DsbC to form ToxR disulfide linked homodimers, through Cys236, leading to transcriptional activity that does not rely on ToxS^29^. However, they were unable to demonstrate chromosomally expressed wild type ToxR formed disulfide linked dimers in the presence of bile salts. Furthermore, they did not demonstrate ToxR-ToxR periplasmic domain interactions without disulfide bonds. In addition, our data suggests the dbToxRp binds ToxSp with a higher affinity than the non-disulfide bonded form, unaffected by bile salts. Consequently, there are two competing models for how ToxR is activated by bile salts. First, the model from Lembke et. al. 2020, where bile salts induce a disulfide bond in the ToxR periplasmic domain, activating transcription that is independent of ToxS^29^. Second, the model put forward in this report, where bile salt induced destabilization is transmitted across the membrane, allowing ToxR to bind DNA augmented by the bound ToxS. As more studies are performed, by us and others, it will be interesting to see what model emerges to explain bile salt induced ToxR activation.

## Materials and Methods

### Cloning of ToxR periplasmic domains

The sequences of the ToxR periplasmic domains from *V. vulnificus, V. parahaemolyticus, V. fischeri, V. harveyi*, and *Photobacterium profundum* were identified by using the ToxR periplasmic sequence from *V. cholerae* (T199-E294). The six ToxR periplasmic domains were codon optimized and synthesized, from 5’ to 3’, with a NcoI site, a 6xHis N-terminal tag followed by the coding sequence, a BamHI site, all flanked by primer sites to amplify the constructs. The constructs were PCR amplified, cut with the appropriate restriction enzymes, and the digested constructs were purified using a PCR clean up kit (Qiagen). The pET16b plasmid was digested with the same restriction enzymes, treated with CIP (NEB), and purified using a PCR clean up kit (Qiagen). The inserts were ligated into the plasmid using the Quick Ligase (NEB) and the reaction was used to transform DH5α’s. Colonies from the transformations were subjected to colony PCR to determine if the plasmids contained an insert of the appropriate size. Then selected colonies were cultured overnight for mini-preps following manufacture instructions (Qiagen). The resulting plasmids were sequence verified using a primer for the T7 promoter.

### Expression and purification

The plasmids with the different ToxR periplasmic domains were used to transform Shuffle T7 Express cells (NEB). The strains were double selected to produce stable expression strains^46^. After double selection the strains were checked for production of soluble protein, by performing a scaled down purification. The ToxR periplasmic domain from *V. vulnificus* was found to produce the greatest amount of soluble protein. Production cultures of *V. vulnificus* and *V. cholerae* ToxR periplasmic domains were started by picking a colony from a freshly streaked plate incubated overnight at 37 °C, and inoculating 2 ml of ZYP^47^ supplements with 0.8% glucose (ZYP-0.8 G) with 200 µg/ml of carbenicillin, and incubated overnight at 30 °C. The next morning the culture was used to inoculate Terrific Broth Modified (Fisher Scientific) media supplemented with 2 mM MgSO_4_ and 200 µg/ml carbenicillin at a ratio of 1:250, then grown to and OD600 of 1–1.2. The cultures were centrifuged at 600 xg for 10 minutes, at 25 °C, with the brake turned off. The cells were resuspended in an equal volume of M9 media with 100 µg/ml carbenicillin and grown for 1 h at 37 °C. The cultures were induced with 1 mM IPTG and incubated overnight at 25 °C with loose covers to allow gas exchange.

For SeMet labeling the frozen culture was restreaked onto a plate containing 200 µg/ml of carbenicillin and incubated overnight at 37 °C. In the morning a single colony was used to inoculate 2 ml ZYP-0.8 G media with 200 µg/ml of carbenicillin and incubated at 37 °C. In the evening the culture was used to inoculate M9 media with 100 µg/ml carbenicillin at 1:12.5 ratio. The culture was grown overnight at 30 °C. The next morning the culture was used to inoculate M9 media at a ratio of 1:20. The culture was incubated at 37 °C till an OD600 of 0.5. Then 25 mg/L of lysine, phenylalanine, and threonine; 12.5 mg/L of isoleucine, leucine, and valine; and 15 mg/L of selenomethionine were added to the culture. The culture was incubated for 15 minutes then induced with 1 mM IPTG and incubated at 25 °C with a loose cover allowing air exchange overnight.

For ^15^N and ^13^C labeling the cultures were started from a single fresh colony in ZYP-0.8 G with 200 µg/ml of carbenicillin overnight at 30 °C. The cultures were used to inoculate TB media at a ratio of 1:250 and incubated at 37 °C till an OD600 of 2. The cultures were centrifuged at 600 xg at 25 °C for 20 minutes with the brake turned off. The supernatant was discarded and the cells were resuspended in an equal volume of M9 media with 100 µg/ml carbenicillin, 3 g/L ^15^N-NH_4_Cl, and 10 g/L of U-^13^C-glucose. The cultures were incubated at 37 °C for 1 h then induced with 1 mM IPTG and incubated at 25 °C overnight.

To purify the protein the cells were harvested by centrifugation at 4500 xg, at 4 °C. The cells were resuspended in lysis buffer (wash buffer (20 mM HEPES pH 7.4, 20 mM imidazole, 200 mM NaCl), a Complete tablet EDTA free (Roche), 3 mM MgSO_4_, 1.5 mM EDTA). The cells were lysed with three passes through a French press. The lysate was clarified by centrifugation at 100,000 xg for 45 minutes. The resulting supernatant was filtered using a 0.45 µm filter before purification. The protein was captured using a His-Trap column (GE Healthcare). The column was washed with 10 CV’s of wash buffer, 10 CV’s of high salt wash buffer (20 mM HEPES pH7.4, 20 mM imidazole, 1 M NaCl), 10 CV’s of wash buffer, followed by 9 CV’s of 9% elution buffer (20 mM HEPES pH 7.4, 500 mM imidazole, 200 mM NaCl). The protein was eluted using a gradient to 100% elution buffer over 4 CV’s with a 2 CV hold at 100%. The relevant fractions were pooled and flowed over an equilibrated thio-propyl sepharose 6B (GE Healthcare) column in order to bind protein with free cysteines. The flow through was collected and concentrated to about 2 ml. The final purification was performed using a S75 16/600 (GE Healthcare) column with the gel filtration buffer (20 mM HEPES pH 7.4, 200 mM NaCl for crystallization and biochemistry, or 20 mM KPO_4_ pH 7.4, 200 mM NaCl for NMR). The relevant fractions were pooled and concentrated as required.

To obtain the non-disulfide bonded form of the ToxR periplasmic domain the thio-propyl column was washed with 2 CV’s of wash buffer, then with 3 CV’s of wash buffer with 100 mM DTT. Two more CV’s of wash buffer with DTT were added to the column and incubated overnight at 16 °C. The following morning the protein was eluted and concentrated to about 2 ml for gel filtration as above.

### Velocity analytical ultracentrifugation

Analytical ultracentrifugation was carried out using Beckman Proteomelab XL-A and an AN-60 rotor. The protein was diluted to an absorbance between 0.3-0.4 in 20 mM HEPES pH 7.4, 200 mM NaCl, 100 µM EDTA. The rotor was run overnight at 30,000 rpm with concentration monitored at 280 nm. The data was processed using SEDFIT with buffer and initial protein parameters calculated with SEDNTERP. The presented sedimentation coefficients and molecular weights are calculated from the major sedimentation peaks representing over 90% of the signal.

### Differential scanning fluorometry

Differential scanning fluorometry was performed to assess the stability of the *V. cholerae* dbToxRp in the presence and absence of additives as described^21^. STATA15 was used to analyze the data. The results are reported from three independent experiments as mean ± standard deviation.

### Pull downs

Pull downs were performed as described^21^. Briefly, the chitin binding domain tagged ToxS periplasmic domain (CBD-ToxSp) was captured on chitin beads from a lysate. After washing the beads purified ToxR periplasmic domain with and without the disulfide bond were added to the tubes with and without 2 mM sodium chenodeoxycholate. After the final wash SDS sample buffer was added to the beads and the tubes were boiled. Samples were diluted and run on a gel with the results quantified and statistics determined using STATA15. The results are from three independent experiments presented as mean ± standard deviation.

### Nuclear magnetic resonance

^1^H,^15^N HSQC^48^ were acquired at 298 K, on a 700 MHz Avance NMR spectrometer equipped with a 5 mm TCI cryoprobe, utilizing 16 scans and 2048 ×256 points. The ToxR periplasmic domain without the disulfide bond was analyzed at a concentration of 580 µM in 20 mM phosphate buffer pH 6.9, 200 mM NaCl, 0.2 mM TCEP, 0.02% NaN_3_, 0.1 mM Pefablock and 1.7% D_2_O. The disulfide bonded ToxR periplasmic domain was analyzed in the 20 mM KPO_4_ pH 7.4, 200 mM NaCl with 1% D_2_O.

### Crystallization and data processing

Crystallization was carried out in a sitting drop with 3 mg/ml of the SeMet labeled *V. vulnificus* intra-chain disulfide bond ToxR periplasmic domain (Vv-dbToxRp) mixed 1:1 with 1.7 M ammonium sulfate, 0.1 M HEPES pH 7.5, and 0.1 M ammonium formate. The well solution with 40% glucose was used as the cryo-protectant. To cryo-protect the crystals the mother liquor was exchanged with the cryo-protectant in the well. Diffraction data was collected at the NSLS2 FMX beam line set at the Se adsorption edge for SAD data collection. The diffraction data was processed in XDS^49^ with a space group of P 2_1_ 2_1_ 2_1_ and unit cell of 39.960 40.530 49.570 90.00 90.00 90.00. The structure was solved using PHENIX Auto-Sol and refined using PHENIX Refine^50^ with COOT^51^ for manual model building. Chimera was used for structure visualization and analysis^34^.

Native Vv-dbToxRp was crystallized by adding in a 1:1 ratio 4 mg/ml of Vv-dbToxRp to 2.0 M ammonium sulfate, and 0.1 M sodium cacodylate pH 6.3 in a sitting drop. Crystals were cryo-protected by dragging the crystal through 75% paratone N and 25% paraffin oil. Data was collected at the NSLS2 FMX beam line. The data was processed using XDS^49^ with the space group P 2_1_ 2_1_ 2_1_ with a of unit cell of 39.99 40.46 50.29 90.00 90.00 90.00. The structure was solved by molecular replacement using PHASER^52^ as implemented in PHENIX^50^, with the previously solved SeMet structure as the search model. The structure was refined using Refine in PHENIX^50^ with manual model building in COOT^51^. Chimera was used for structure visualization and analysis^34^.

## Supplementary information

Supplementary information.
