# Publisher Correction: A disulfide constrains the ToxR periplasmic domain structure, altering its interactions with ToxS and bile-salts

**DOI:** 10.1038/s41598-020-69095-8

**Published:** 2020-07-16

**Authors:** Charles R. Midgett, Rachel A. Swindell, Maria Pellegrini, F. Jon Kull

**Affiliations:** Department of Chemistry, Dartmouth College, Hanover, NH USA

Correction to: *Scientific Reports* https://doi.org/10.1038/s41598-020-66050-5, published online 02 June 2020

In this Article, an additional information section was omitted. This should read:

“All strains, plasmids, and protocols are available upon request. The coordinates for both structures have been deposited in the PDB: SeMet-Vv-dbToxRp, 6uue; native Vv-dbToxRp, 6utc.”

