# Supplementary material for: A disulfide constrains the ToxR periplasmic domain structure, altering its interactions with ToxS and bile-salts
Source: Sci Rep. 2020 Jun 2;10:9002. doi: 10.1038/s41598-020-66050-5 (PMC7265457; doi:10.1038/s41598-020-66050-5)
Supplement: Supplementary file 1 — Supplementary information. [file 41598_2020_66050_MOESM1_ESM.pdf]

Supplementary information:

A disulfide constrains the ToxR periplasmic domain structure, altering its interactions with ToxS and bile-salts

Charles R Midgett[1]*, Rachel A Swindell[1], Maria Pellegrini[1], and F Jon Kull[1]

[1]Department of Chemistry, Dartmouth College, Hanover, NH

*Corresponding Author: Charles R Midgett charles.midgett@dartmouth.edu

Rachel A Swindell swindellra@gmail.com

Maria Pellegrini maria.pellegrini@dartmouth.edu

F Jon Kull f.jon.kull@dartmouth.edu

Figure S1:

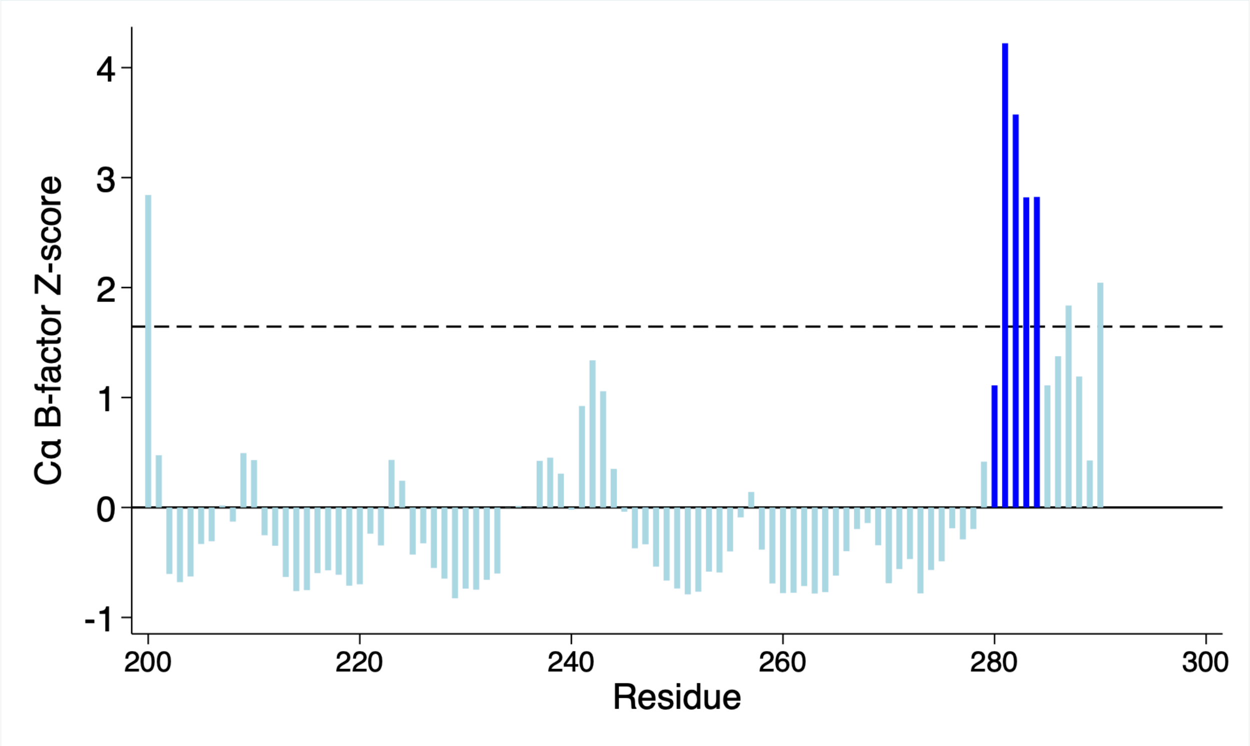

Figure S1: Graph of B-factor Z-score of the Cα at each amino acid. The Cα B-factors were standardized and graphed using STATA 15. The Z-score of the β5-α2 loop residues Cα's, numbered 280-284, are shown as blue lines while remaining Z-scores are depicted as light blue lines. The dotted line corresponds to a Z-score at .05 in the distribution. As can be seen the Cα Z-scores for residues 281-284 are within the top .05 of the distribution and among the highest in the structure.

Figure S2:

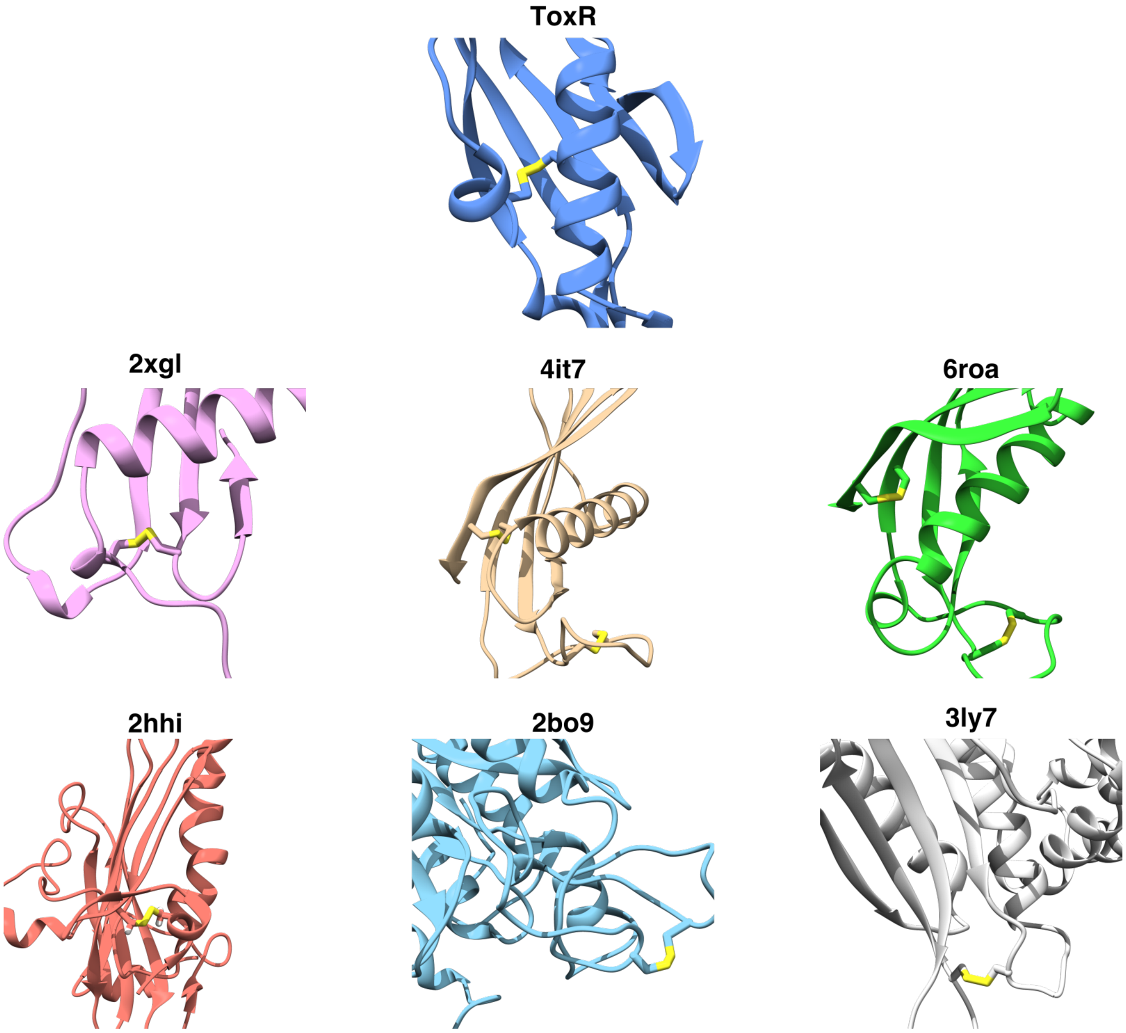

Figure S2: Disulfide bonds in the proteins identified in the DALI search. The cysteine bonds are displayed as sticks for each protein. The ToxR periplasmic domain is displayed at the top and the other proteins from the PDB are displayed below. In the ToxR periplasmic domain the disulfide bond is between the two α-helices and the disulfide bonds in the other proteins are between different structural elements.

Figure S3:

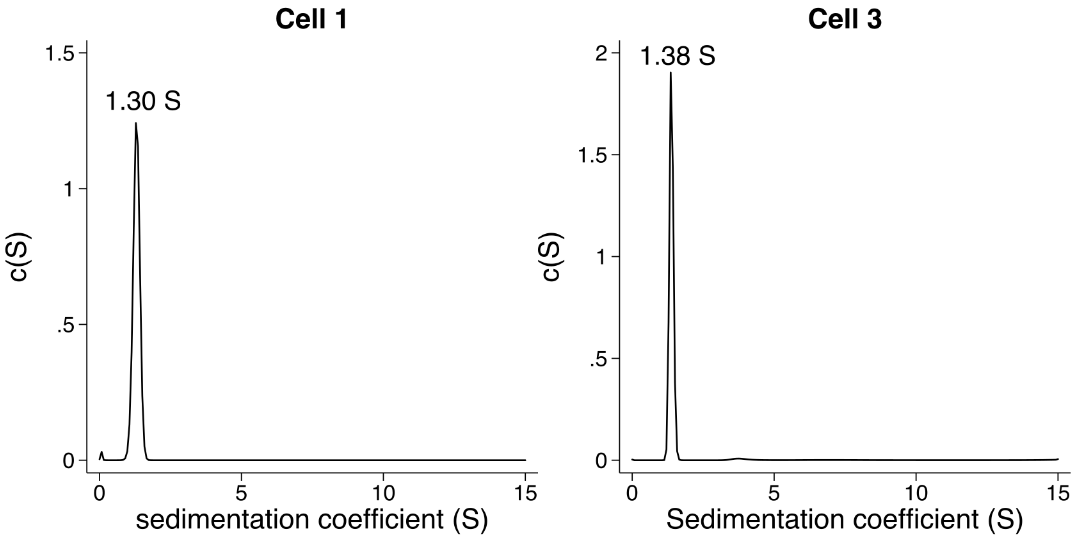

Figure S3: Results from the analytical ultracentrifugation of Vc-dbToxRp. Graphs from each cell with the sedimentation coefficient for each peak. In cell 1 the calculated molecular weight was 11.8 kDa and in cell 3 the calculated molecular weight was 12.7 kDa. The molecular weight of the domain is 11.5 kDa, therefore the dbToxRp is a monomer in solution.

Figure S4:

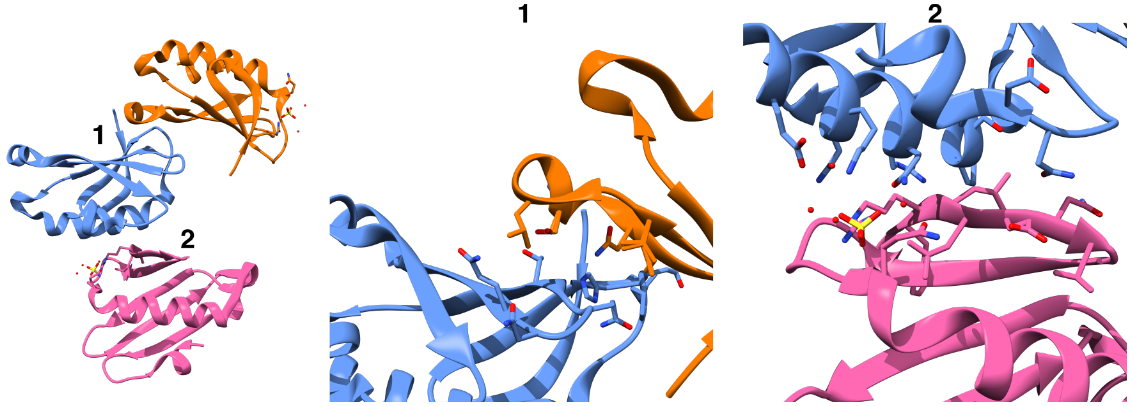

Figure S4: Figure showing crystal contacts that were explored as potential dimerization interfaces. Left panel shows how the monomers contact each other in the crystal. Detail of the first (middle panel) and the second (right panel) interface. Note there are few hydrogen bonds between the potential interfaces and in interface 2 the sulfate ion appears to be stabilizing the interface.

Figure S5

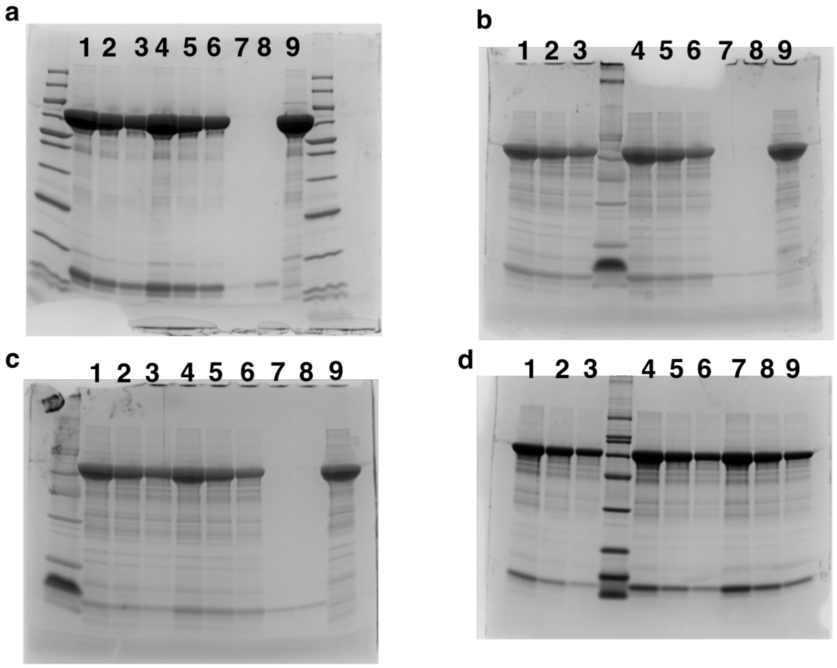

Figure S5: Gels of the pull downs excerpted in figure 1 and 2. **A** is the gel from the validation that the dbToxRp is pulled down by ToxS. Lanes 1-3 are the 1x, 1/2x, and 1/4x dilutions of the non-disulfide containing ToxR periplasmic domain. Lanes 4-6 are the dilution series of the dbToxRp pull down. Lane 7 is the dbToxRp only control, lane 8 is the ToxRp only control, and lane 9 is the ToxSp lysate only control. **B** is the gel of the dbToxRp pull down with and without CDC. Lanes 1-3 are the 1x, 1/2x, 1/4x dilution series of dbToxRp without CDC and lanes 4-6 are the dilution series of dbToxRp with CDC. Lane 8 is the dbToxRp only control without CDC, lane 9 is the dbToxRp control with CDC and lane 10 is CBDI-ToxSp only control. **C** is the ToxRp pull down with and without CDC, lanes 1-3 is the buffer treated dilution series, lanes 4-6 are the CDC treated dilution series, lane 7 is the ToxRp only control, lane 8 is the ToxRp with CDC control, and lane 9 is the CBDI-ToxSp only control. **D** is the gel comparing the amount of ToxRp pulled down relative to dbToxRp. Lanes 1-3 is the dilution series of ToxRp with buffer, and lanes 4-6 is the dilution series of ToxRp with CDC, and lanes 7-9 is the dilution series of dbToxRp with CDC.

Table S1 Refinement Statistics

	Native_VvToxRp	SeMet-VvToxRp
Wavelength (Å)	0.97933	0.97934
Resolution range	28.43 - 1.249 (1.294 - 1.249)	24.78 - 1.389 (1.439 - 1.389)
Space group	P 21 21 21	P 21 21 21
Unit cell	39.97 40.44 50.28 90 90 90	39.96 40.53 49.57 90 90 90
Total reflections	229089 (5713)	202709 (13693)
Unique reflections	20417 (1084)	16768 (1604)
Multiplicity	11.2 (5.3)	12.1 (8.5)
Completeness (%)	87.81 (47.08)	98.73 (92.82)
Anomalous Completeness (%)		99.66
Mean I/sigma(I)	23.88 (1.16)	21.66 (1.20)
Wilson B-factor	17.28	21.45
R-merge	0.0473 (0.9164)	0.06322 (1.182)
R-meas	0.04944 (1.016)	0.06591 (1.258)
R-pim	0.0141 (0.4206)	0.0184 (0.4185)
CC1/2	1 (0.446)	0.999 (0.636)
CC*	1 (0.786)	1 (0.882)
Reflections used in refinement	20408 (1081)	16585 (1512)
Reflections used for R-free	1980 (108)	1654 (149)
R-work	0.1955 (0.2853)	0.2007 (0.2863)
R-free	0.2118 (0.2857)	0.2287 (0.3124)
CC(work)	0.964 (0.721)	0.965 (0.819)
CC(free)	0.955 (0.684)	0.963 (0.809)
Number of non-hydrogen atoms	848	770
macromolecules	737	708
ligands	5	5
solvent	106	57
Protein residues	91	91
RMS(bonds)	0.006	0.006
RMS(angles)	0.86	0.81
Ramachandran favored (%)	96.63	98.88
Ramachandran allowed (%)	3.37	1.12
Ramachandran outliers (%)	0	0
Rotamer outliers (%)	2.35	0
Clashscore	4.73	2.82
Average B-factor	23.7	27.72
macromolecules	22.44	27.05
ligands	23.34	27.76
solvent	32.5	35.96

Table S2

					DALI Results				Chimera MatchMaker Results	
No:	Curated No:	Chain	Z-score	RMSD	No: residues aligned	Chain length	%id PDB	Description	No: residues aligned	RMSD
1	1	1vcy-A	5.4	2.5	69	193	9	MOLECULE: VOLVATOXIN A2;	80	18.6
2	2	2na4-A	5.2	2.5	62	108	5	MOLECULE: CURLI PRODUCTION ASSEMBLY/TRANSPORT COMPONENT CSG	64	7.5
6	3	4hei-B	4.6	2.8	65	90	5	MOLECULE: RIBOSOME HIBERNATION PROTEIN YHBH;	72	7.0
8	4	2ftk-C	4.5	3.6	73	181	14	MOLECULE: SPORULATION INITIATION PHOSPHOTRANSFERASE B;	75	12.1
11	5	4r80-B	4.5	2.8	62	76	13	MOLECULE: OR486;	50	13.8
13	6	2hhi-A	4.3	3.2	74	204	5	MOLECULE: IMMUNOGENIC PROTEIN MPT64;	82	13.9
16	7	5x8r-y	4.3	3.1	75	108	7	MOLECULE: 30S RIBOSOMAL PROTEIN S2, CHLOROPLASTIC;	67	8.7
18	8	4htg-A	4.3	3.3	71	302	13	MOLECULE: PORPHOBILINOGEN DEAMINASE, CHLOROPLASTIC;	82	13.0
25	9	2ywq-B	4.2	2.9	65	89	3	MOLECULE: RIBOSOMAL SUBUNIT INTERFACE PROTEIN;	70	7.7
26	10	3b4r-A	4.1	3.0	68	218	6	MOLECULE: PUTATIVE ZINC METALLOPROTEASE MJ0392;	74	15.7
27	11	5coz-A	4.1	3.5	75	347	5	MOLECULE: UNCHARACTERIZED PROTEIN;	82	23.1
30	12	3zxo-B	4.1	3.3	67	125	7	MOLECULE: REDOX SENSOR HISTIDINE KINASE RESPONSE REGULATOR	71	13.8
32	13	2owp-A	4.1	2.8	63	129	6	MOLECULE: HYPOTHETICAL PROTEIN BXE_B1374;	61	18.5
35	14	1ixm-B	4.1	3.4	69	175	12	MOLECULE: PROTEIN (SPORULATION RESPONSE REGULATORY	74	11.1
38	15	3f7s-A	4.0	2.8	63	142	10	MOLECULE: UNCHARACTERIZED NTF2-LIKE PROTEIN;	67	16.5
49	16	4r3a-A	3.9	3.8	72	318	14	MOLECULE: BLUE-LIGHT-ACTIVATED HISTIDINE KINASE 2;	75	12.6
50	17	1n3g-A	3.9	2.9	67	113	4	MOLECULE: PROTEIN YFIA;	65	7.4

52	18	2rcd-B	3.9	4.0	67	128	7	MOLECULE: UNCHARACTERIZED PROTEIN;	62	18.3
60	19	3tqm-C	3.9	2.9	65	93	9	MOLECULE: RIBOSOME-ASSOCIATED FACTOR Y;	68	7.1
63	20	2m7o-A	3.8	2.5	54	70	9	MOLECULE: UNCHARACTERIZED PROTEIN;	56	16.9
64	21	1imu-A	3.8	2.7	66	107	5	MOLECULE: HYPOTHETICAL PROTEIN HI0257;	66	7.1
70	22	3k0z-B	3.8	2.8	65	149	6	MOLECULE: PUTATIVE POLYKETIDE CYCLASE;	69	17.6
71	23	4u89-A	3.8	3.4	69	225	6	MOLECULE: PHOSPHOPANTETHEINYL TRANSFERASE PPTT;	78	14.2
72	24	6bjt-B	3.7	3.6	66	130	9	MOLECULE: DUF4440 DOMAIN-CONTAINING PROTEIN;	62	16.8
82	25	1pp6-A	3.7	2.8	69	194	9	MOLECULE: VOLVATOXIN A2;	79	18.3
83	26	4it7-C	3.7	3.7	65	107	6	MOLECULE: CPI;	57	15.2
89	27	2xft-B	3.7	3.4	66	424	11	MOLECULE: ORF12;	75	29.3
93	28	6ct5-A	3.7	3.5	69	220	6	MOLECULE: 4'-PHOSPHOPANTETHEINYL TRANSFERASE;	74	17.2
96	29	4gt8-A	3.7	3.5	68	133	10	MOLECULE: SENSOR PROTEIN VRAS;	78	11.1
97	30	2bo9-D	3.7	3.6	63	217	8	MOLECULE: CARBOXYPEPTIDASE A4;	73	18.2
98	31	5iw9-A	3.6	3.0	57	126	9	MOLECULE: BASEPLATE WEDGE PROTEIN GP25;	61	8.9
99	32	6e52-A	3.6	3.6	68	243	6	MOLECULE: STAPHYLOCOCCUS AUREUS AGRC HISTIDINE KINASE MODULE	74	14.3
101	33	2xgl-A	3.6	3.0	60	90	2	MOLECULE: COLICIN-M IMMUNITY PROTEIN;	57	16.3
105	34	4fr9-A	3.6	3.0	53	141	6	MOLECULE: UNCHARACTERIZED PROTEIN;	76	15.0
109	35	3k7c-B	3.6	2.7	59	108	5	MOLECULE: PUTATIVE NTF2-LIKE TRANSPEPTIDASE;	64	15.9
112	36	6gve-D	3.6	1.8	52	337	8	MOLECULE: CP12 POLYPEPTIDE;	76	17.7
116	37	6roa-A	3.6	3.3	64	109	8	MOLECULE: CYSTATIN-C;	66	14.8

118	38	5kew-E	3.6	3.0	65	88	8	MOLECULE: VTRA PROTEIN;	75	5.5

Table S1: Table showing excerpted curated DALI and Chimera MatchMaker results of the Vv-dbToxRp structure. Highlighted in yellow is the alignment with the VtrA structure
